# No association between early antiretroviral therapy during pregnancy and plasma levels of angiogenic factors: a cohort study

**DOI:** 10.1186/s12884-019-2600-4

**Published:** 2019-12-09

**Authors:** Ameyo Djeha, Sylvie Girard, Helen Trottier, Fatima Kakkar, Hugo Soudeyns, Marc Boucher, Normand Lapointe, Isabelle Boucoiran

**Affiliations:** 1 Department of Obstetrics and Gynecology, Faculty of Medicine, Université de Montréal, Montreal, Canada; 2 Department of Social and Preventive Medicine, Faculty of Medicine, Université de Montréal, Montreal, Canada; 3 Centre de recherche du CHU Sainte-Justine, Montreal, Canada; 4 Centre d’infectiologie Mère-Enfant, CHU Sainte-Justine, Montreal, Canada; 5 Division of Infectious Diseases, CHU Sainte-Justine, Montreal, Canada; 6 Department of Pediatrics, Faculty of Medicine, Université de Montréal, Montreal, Canada; 7 Unité d’immunopathologie virale, Centre de recherche du CHU Sainte-Justine, Montreal, Canada; 8 Department of Microbiology, Infectiology & Immunology, Faculty of Medicine, Université de Montréal, Montreal, Canada; 9 Department of Obstetrics and Gynecology, Centre hospitalier universitaire (CHU) Sainte-Justine, 3175 Côte Sainte-Catherine, Montreal, QC H3T 1C5 Canada

**Keywords:** Antiretroviral therapy, HIV, Placental function, Placental growth factor, Soluble fms-like tyrosine kinase-1

## Abstract

**Background:**

Early antiretroviral therapy (ART) during pregnancy has dramatically reduced the risk of perinatal HIV transmission. However, studies have shown an association between premature delivery and the use of ART during pregnancy (particularly protease inhibitor (PI)-based therapies), which could be explained by placental dysfunction. The objective of this study was to evaluate the association of ART (class, duration of exposure and time of initiation) with placental function by using angiogenic factors placental growth factor (PlGF) and soluble fms-like tyrosine kinase-1 (sFlt-1) as biomarkers.

**Methods:**

Clinical and biological data from 159 pregnant women living with HIV were analyzed. Levels of each biomarker were measured in the first and second trimester of pregnancy. After logarithmic transformation, we compared these using generalized estimating equations according to (a) the type of ART; (b) the duration of exposure to ART; and (c) the time of initiation of ART.

**Results:**

After adjusting for variables such as ethnicity, maternal age, gestational age, body mass index, parity, smoking status, and sex of the fetus, we found no significant association between the class of ART (PI-based or not) and serum concentrations of PlGF or sFlt-1. Furthermore, no significant association was found between biomarker levels and the duration of ART exposure or the timing of ART initiation (pre- or post-conception).

**Conclusions:**

This study suggests that first and second trimester angiogenic factor levels are not significantly associated with ART, regardless of the duration or type (with or without PI). These observations seem reassuring when considering the use of ART during early pregnancy.

## Background

An estimated 17.8 million of the 36.7 million people living with HIV in 2016 (48.5%) were women of childbearing age (15 years and older) [1]. Despite their HIV status, many of these women want to have children [2]. Antiretroviral therapy (ART) reduces the risk of HIV transmission from mother to child to 1–2%, compared to 15–40% without intervention [3]. ART also improves the health status of the mothers, raises their quality of life and prolongs their life expectancy, leading more and more women living with HIV to conceive [2]. According to current recommendations, all adults living with HIV should be treated with ART [4–7]. As a result, most women living with HIV are now already on ART at the time of conception and early pregnancy, whereas in the past, ART was initiated only in the second trimester of pregnancy [8].

Although ART has significantly reduced rates of adverse perinatal outcomes such as stillbirth, intrauterine growth restriction and preterm birth associated with maternal HIV infection [9, 10], these rates are still higher for women living with HIV while receiving ART compared to HIV-negative women [11–14]. These adverse pregnancy outcomes may be related to the class of ART used during pregnancy, notably to protease inhibitor (PI) regimens [14–18], which have been associated with placental vascular changes [19].

Anti-angiogenic effects have been reported following the use of PIs in oncology [20, 21]. Placental growth factor (PlGF) and the soluble receptor, soluble fms-like tyrosine kinase-1 (sFlt-1) are respectively pro- and anti-angiogenic factors. PlGF is synthesized in several organs (heart, skeletal muscle, lungs, adipose tissue, platelets). sFlt-1 is released by vascular endothelial cells and circulating cells (monocytes, macrophages, platelets). The placenta (trophoblast and endothelial cells of the placental villi) is the main source of PlGF and sFlt-1 during pregnancy; they participate in vasculogenesis and feto-placental angiogenesis. Increased sFlt-1 and decreased free PlGF levels in maternal blood, or increased sFlt-1/ PlGF ratio are directly implicated in the pathophysiology of preeclampsia, including maternal endothelial dysfunction [22–29]. A change in these factors is also associated with other complications during pregnancy, such as intrauterine growth restriction [27, 30], preterm delivery [31], spontaneous abortion and stillbirth [26, 32, 33], ,confirming the association between impaired placental perfusion and systemic changes in angiogenic factors [34–36]. These observations have been noted in cohorts of women living with HIV [37].

We hypothesized that pregnant women living with HIV who receive PI-based ART may have impaired placental function compared to untreated women living with HIV, altering the plasma concentration of the angiogenic factors placental growth factor (PlGF) and soluble fms-like tyrosine kinase-1 (sFlt-1). These disturbances could be influenced by the duration of ART exposure or by the timing of ART initiation. The objective of this study was to evaluate the association of the class, duration and timing of initiation of ART with serum concentrations of PlGF and sFlt-1 biomarkers, in the first and second trimesters of pregnancy.

## Methods

This study used data from the database of pregnant women living with HIV who were consented and enrolled at their first antenatal visit in the prospective cohort of Centre maternel et infantile sur le sida (CMIS), CHU Sainte-Justine, Montreal, QC, Canada. The CMIS database and biobank contain information and biologic samples from more than 900 mother-child pairs and enrolls approximately 40 additional pairs annually. De-identified data are collected and managed using REDCap electronic data capture tools [38].

This study included data from pregnant women who were enrolled between January 2003 and December 2016, and for whom serum samples were available during both the first trimester (5–14 weeks) and the second trimester (15–28 weeks) of pregnancy. Maternal serum samples were collected at the time of clinically indicated blood tests and stored at − 80 °C for research purpose. Levels of PlGF and sFlt-1 were measured using DuoSet Enzyme-Linked Immunosorbent Assay (ELISA) kits (R&D systems, Minneapolis, MN). Lower limits of detection were 15.6 pg/ml (sFlt-1) and 62.5 pg/ml (PlGF). There was no dilution for PlGF and it is a factor of 5 for sFLT-1. In all cases, both biomarkers were assayed using the same serum aliquot.

Gestational age was defined based on the crown-rump length from the first-trimester ultrasound if available and if not, from the date of the last menstrual period. Preterm birth was defined as delivery *before* 37 weeks of gestation. Small for gestational age (SGA) was defined as a birth weight below the 10th percentile for the gestational age.

Women living with HIV were categorized according to ART exposure at first and second trimester (PI-based ART, other ART, or no treatment). The duration of ART exposure during pregnancy was expressed in *weeks since conception*. Time of initiation of ART was defined relative to *conception* (either before conception or during pregnancy).

### Statistical analysis

Descriptive analyses were conducted on the socio-demographic, clinical and biological data of the participants. For each categorical and continuous variable, data are reported as proportions or mean (with standard deviation) or median with interquartile range (IQR) respectively. The Wilcoxon test for matched samples was used to compare serum marker concentrations in the first and second trimesters and Mann-Withney U test to compare angiogenic factor levels in the two groups with undetectable viral load or not.

Linear regression evaluated the association between angiogenic factor concentrations and birth outcome groups (preterm birth and SGA) at the first and second trimester. To account for repeated measurements from the same individuals in the first and second trimesters of pregnancy, linear generalized estimating equations (GEE) were used to analyze the association between ART (class, duration of exposure and initiation time) and plasma concentration of the two biomarkers. A first-order autoregressive (AR1) correlation matrix was used. Models were adjusted for potential confounding factors previously identified in a review of the literature, including ethnicity, parity, maternal age, gestational age, body mass index (BMI), smoking status and sex of the fetus [39–43]. Confounding variables that resulted in a +/− 10% variation of the regression coefficient when introduced into the bivariate model were retained in the final model. All variables with a *p* < 0.05 were also included in the final model. As suggested by residual analysis, a logarithmic transformation of biomarkers levels was performed. Using sensitivity analyses, we considered models with different unstructured or exchangeable correlation matrices and compared these models using Quasi-likelihood under Independence Model Criterion (QIC). A value of *p* < 0.05 was considered statistically significant. 95% confidence intervals are shown. Considering the values of PlGF and sFlt-1 (mean and standard deviation) obtained in the reference group (group without ART), we calculated that with the available sample size (*n* = 159), we could have been able to detect respective mean (delta) differences of 33.67 and 1905.15 pg / ml using an alpha error of 0.05 and a statistical power (beta) of 80%. Statistical analyses were performed using IBM SPSS Statistics for Windows version 24 (IBM Corp, Armonk, NY).

The use of the CMIS database and biobank for this study was approved by the directors of the CMIS (FK and NL) and CHU Sainte-Justine ethics review board. All participants provided written informed consent.

## Results

A total of 318 paired serum samples from 159 pregnant women living with HIV were analyzed. Demographic, clinical and biological characteristics of the participants are presented in Table 1. In the first trimester, nearly 69% of women were receiving ART, 82% of whom were receiving PI-based regimens. In the second trimester, more than 96% were receiving ART, 86% of which were PI-based. The mean duration of ART exposure from the start of pregnancy to first-trimester and second-trimester biomarker testing was 9.5 ± 2.9 weeks and 14.8 ± 7.1 weeks, respectively.

**Table 1:** Population characteristics

Characteristics	1st trimester *n* = 159			2nd trimester *n* = 159			Total *n* = 159
	No ART *n* = 50	PI *n* = 89	Other regimens *n* = 20	No ART *n* = 6	PI *n* = 131	Other regimens *n* = 22	
Maternal age at delivery, years, mean ± SD	31.7 ± 5.3	33.1 ± 4.9	31.3 ± 5.0	29.7 ± 3.0	32.5 ± 5.1	32.3 ± 5.4	32.4 ± 5.1
Ethnicity, n (%)							
Afro-Caribbean	43 (86.0)	69 (77.5)	18 (90.0)	6 (100.0)	103 (78.6)	21 (95.5)	130 (81.8)
Caucasian	7 (14.0)	17 (19.1)	1 (5.0)	0 (0.0)	24 (18.3)	1 (4.5)	25 (15.7)
Other	0 (0.0)	3 (3.4)	1 (5.0)	0 (0.0)	0 (0.0)	0 (0.0)	4 (2.5)
BMI, kg/m^2^, median [IQR]	27.2 [24.9–31.0]	25.8 [22.8–29.9]	26.5 [22.9–30.3]	28.2 [24.4–34.9]	26.4 [23.6–30.4]	24.9 [23.2–28.7]	26.3 [23.5–30.2]
Parity, n (%)							
0	15 (30.0)	31 (34.8)	6 (30.0)	2 (33.3)	41 (31.3)	9 (40.9)	52 (32.7)
1	18 (36.0)	31 (34.8)	10 (50.0)	4 (66.7)	45 (34.4)	10 (45.5)	59 (37.1)
2	12 (24.0)	14 (15.7)	1 (5.0)	0 (0.0)	26 (19.8)	1 (4.5)	27 (17.0)
3 and more	5 (10)	13 (14.7)	3 (15.0)	0 (0.0)	19 (14.5)	2 (9.1)	21 (13.2)
Smoker, n (%)	6 (12.0)	8 (9.0)	1 (5.0)	0 (0.0)	6 (4.6)	0 (0.0)	
Chronic hypertension, n (%)	2 (4.0)	1 (1.1)	0 (0.0)	0 (0.0)	3 (2.3)	0 (0.0)	3 (1.9)
Gestational hypertension or preeclampsia, n (%)	4 (8.0)	9 (10.1)	2 (10.0)	1 (16.7)	11 (7.2)	3 (13.6)	15 (9.4)
Sex of fetus, n (%)							
Male	25 (50.0)	53 (59.6)	4 (20.0)	1 (16.7)	76 (58.0)	5 (22.7)	82 (51.6)
CD4 count^a^, median [IQR]							
Total/mm^3^	495.5 [270.0–679.5]	573.5 [404.25–751.5]	580.5 [372.5–733.5]	493.5 [193.5–859.5]	561.0 [378.5–722.5]	645.0 [470.75–1056.0]	
Percentage	25.0 [16.0–33.0]	32.0 [25.0–38.5]	33.5 [23.0–43.5]	26.0 [17.0–37.0]	31.0 [24.0–39.0]	40.0 [27.0–44.0]	
Undetectable viral load^a^, n (%)	4 (8.0)	72 (80.9)	16 (80.0)	2 (33.3)	94 (71.8)	19 (86.4)	
Pregnancy outcome, n (%)							
SGA	8 (16.0)	19 (21.3)	2 (10.0)	1 (16.7)	26 (19.8)	2 (9.1)	29 (18.6)
Preterm birth	6 (12.0)	17 (19.1)	2 (10.0)	0 (0.0)	22 (16.8)	3 (13.6)	25 (15.7)
Gestationnal age^a^ weeks, median [IQR]	9.7 [7.9–11.9]	10.7 [8.9–11.9]	10.7 [7.9–11.8]	18.3 [15.8–19.1]	19.6 [17.1–21.3]	19.3 [17.1–23.0]	

*SD* Standard deviation, *BMI* Body mass index, *IQR* Interquartile range, *ART* Antiretroviral therapy, *SGA* Small for gestational age

^a^At sampling

The median concentration of PlGF in the first trimester was 93.5 pg/ml [IQR = 74.2–129.0] compared to 229.0 pg/ml [IQR = 154.8–329.0] in the 2nd trimester (*p* < 0.0001). The median concentration of sFlt-1 in the first trimester was 3372.7 pg/ml [IQR = 1736.7–5781.8] compared to 4009.1 pg/ml [IQR = 2600.0–8236.4] in the second trimester (*p* = 0.006). The angiogenic factors levels are similar in the two groups with undetectable viral load or not in the first trimester (respectively 93.5 pg/ml [IQR = 74.2–125.0] compared to 91.9 pg/ml [IQR = 77.4–145.2]; *p* = 0.752 for PlGF and 3668.2 pg/ml [IQR = 1452.3–7509.1] compared to 2850.0 pg/ml [IQR = 1963.6–5554.5]; *p* = 0.194 for sFlt-1) and in the second trimester (respectively 229.0 pg/ml [IQR = 148.4–322.6] compared to 240.3 pg/ml [IQR = 177.4–392.7]; *p* = 0.328 for PlGF and 4145.5 pg/ml [IQR = 2600.0–10,190.9] compared to 3918.2 pg/ml [IQR = 2611.4–6725.0]; *p* = 0.659 for sFlt-1).

**Fig. 1:**
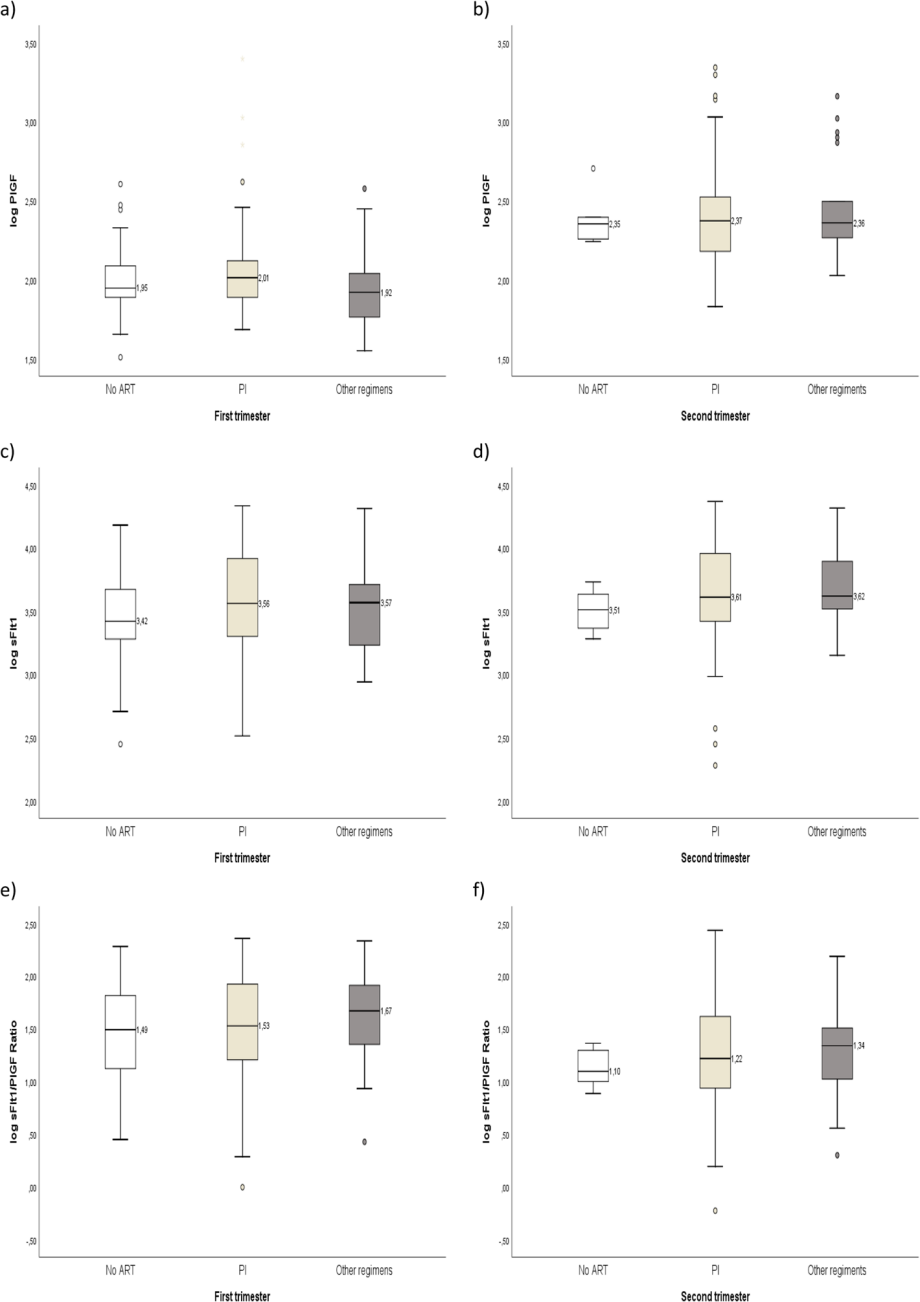
Biomarker distribution by class of antiretroviral therapy (Box-and-whiskers representation). **a** Placental growth factor level (PlGF) in 1st trimester; **b** PlGF level in 2nd trimester; **c** soluble fms-like tyrosine kinase-1 (sFlt-1) level in 1st trimester; **d** sFlt-1 level in 2nd trimester **e** sFlt-1/PlGF ratio in ^1st^ trimester; **f** sFlt-1/PlGF ratio in 2nd trimester. First trimester: no ART, *n* = 50; PI, *n* = 89, other regimens, *n* = 20; Second trimester: no ART, *n* = 6; PI, *n* = 131, other regimens, *n* = 22. The boxes extend from the 25th percentile to the 75th percentile (i.e., the interquartile range); lines inside boxes represent median values. Lines emerging from boxes (i.e., the whiskers) extend to the upper and lower adjacent values. The lower adjacent values provide an estimate of the lower limit of the array and represents the first quartile value less 1.5 times the difference between the first and third quartiles. The upper adjacent value provides an estimate of the upper limit of the array and represents the third quartile value plus 1.5 times the difference between the first and third quartiles. Values outside these limits are outliers. ART: antiretroviral therapy; PI: protease inhibitor

**Fig. 2:**
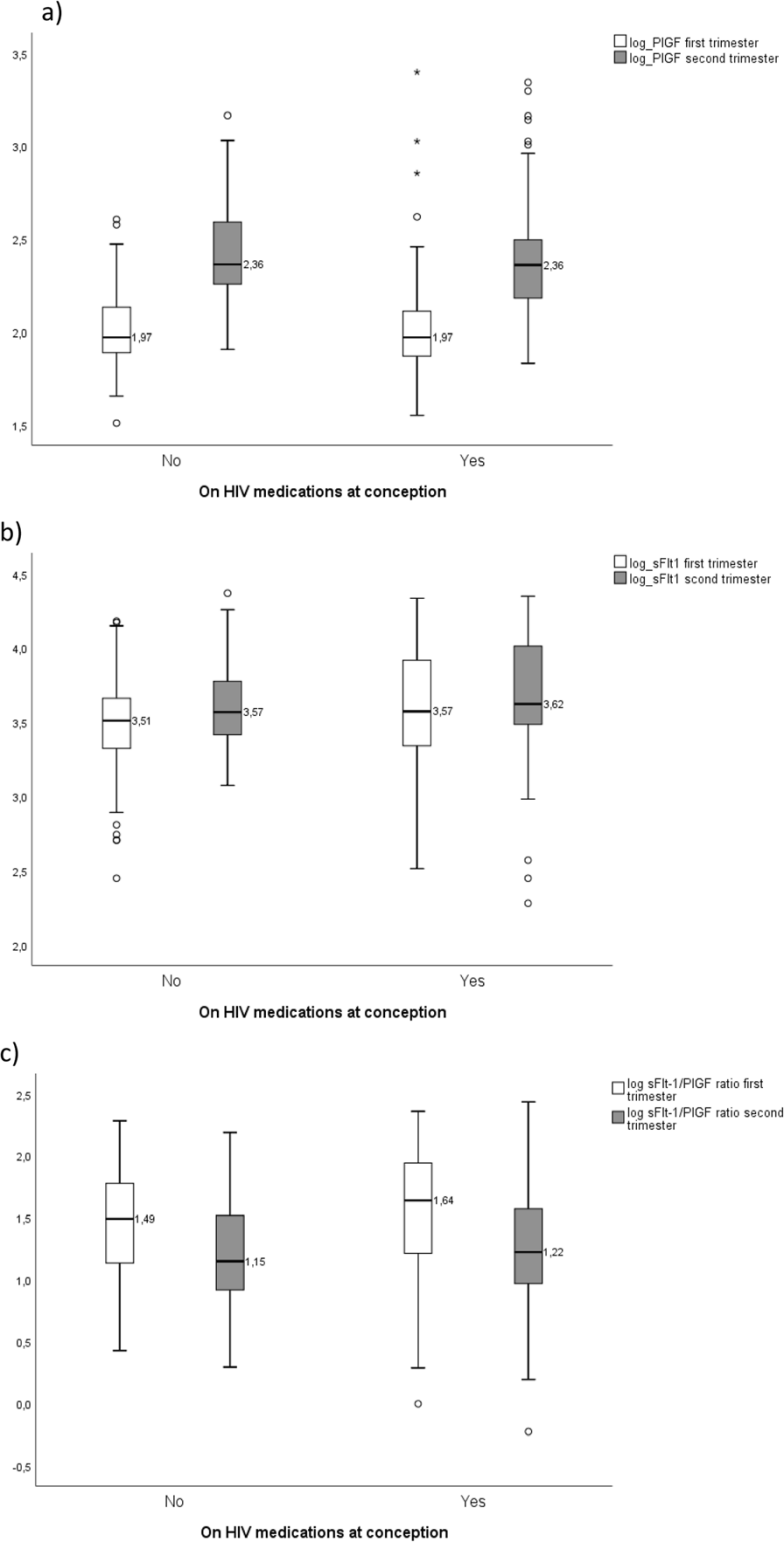
Biomarker distribution by time of ART initiation (Box-and-whiskers representation). **a** Placental growth factor level (PlGF) in 1st trimester and 2nd trimester; **b** soluble fms-like tyrosine kinase-1 (sFlt-1) level in 1st trimester and 2nd trimester **c** sFlt-1/PlGF ratio in ^1st^ trimester and 2nd trimester. By time of ART initiation (before or after conception). On HIV medication at conception: No, *n* = 62; Yes, *n* = 97. The boxes extend from the 25th percentile to the 75th percentile (i.e., the interquartile range); lines inside boxes represent median values. Lines emerging from boxes (i.e., the whiskers) extend to the upper and lower adjacent values. The lower adjacent values provide an estimate of the lower limit of the array and represents the first quartile value less 1.5 times the difference between the first and third quartiles. The upper adjacent value provides an estimate of the upper limit of the array and represents the third quartile value plus 1.5 times the difference between the first and third quartiles. Values outside these limits are outliers. ART: antiretroviral therapy; PI: protease inhibitor

Figure 1 shows the distribution of the different biomarkers according to the class of ART and Fig. 2 the distribution by time of ART initiation.

Bivariate comparisons are presented in Table 2. After adjustment, no significant association was found between the class of ART (whether PI-based or not) and the level of PlGF, sFlt-1 or the PlGF/SFlt-1 ratio (Table 2). Furthermore, no significant association was found between serum angiogenic factors and duration of ART exposure (including all PI-containing ART) or initiation time (pre-conception versus during pregnancy) (Table 2).

**Table 2:** Association between biomarkers and ART (type, duration and initiation time)

Biomarkers	Crude coefficient	CI (95%)	*p*-value	Adjusted coefficient^a^	Adjusted CI (95%)	*p*-value
PlGF						
PI-based ART^b^	0.238	0.166–0.310	0.000	0.018^c^	− 0.051 - 0.088	0.611
Other ART^b^	0.201	0.095–0.307	0.000	0.028^c^	− 0.064 - 0.121	0.552
ART exposure duration	0.020	0.014–0.026	0.000	0.001^d^	− 0.006 - 0.007	0.873
PI exposure duration	0.018	0.011–0.025	0.000	0.000^d^	−0.007 - 0.006	0.936
Time of ART initiation^e^	−0.019	− 0.089 - 0.051	0.592	0.000^d^	−0.066 - 0.066	0.997
sFlt1						
PI-based ART^b^	0.306	0.109–0.503	0.002	0.177^f^	− 0.032 - 0.386	0.097
Other ART^b^	0.233	−0.108 - 0.573	0.180	0.091^f^	−0.246 - 0.428	0.597
ART exposure duration	0.003	−0.008 - 0.015	0.584	−0.001^g^	−0.014 - 0.012	0.872
PI exposure duration	0.007	−0.004 - 0.019	0.218	0.002^g^	−0.010 - 0.015	0.722
Time of ART initiation^e^	0.085	−0.074 - 0.244	0.294	0.024 ^h^	−0.139 - 0.188	0.772
sFlt1/PlGF Ratio						
PI-based ART^b^	0.033	−0.179 - 0.245	0.759	0.145^i^	−0.076 - 0.366	0.199
Other ART^b^	−0.036	−0.386 - 0.314	0.840	0.063^i^	−0.319 - 0.445	0.747
ART exposure duration	−0.014	−0.028 - 0.000	0.044	0.000^f^	−0.018 - 0.017	0.956
PI exposure duration	−0.007	−0.021 - 0.006	0.299	0.003^h^	−0.013 - 0.019	0.683
Time of ART initiation^e^	0.104	−0.071 - 0.279	0.243	0.028^h^	−0.152 - 0.208	0.761

*ART* Antiretroviral therapy, *PI* Protease inhibitor, *CI* Confidence interval

^a^ Linear Generalized Estimating Equations

^b^ Control: no ART

^c^ Adjusted for gestational age at the date of test and ethnicity

^d^ Adjusted for gestational age at the date of test, body mass index and ethnicity

^e^ Control: ART initiated during pregnancy

^f^ Adjusted for gestational age at the date of test and body mass index

^g^ Adjusted for gestational age at the date of test and maternal age

^h^ Adjusted for gestational age at the date of test, maternal age and ethnicity

^i^ Adjusted for gestational age at the date of test, maternal age, body mass index, parity, and sex of the fetus

The association between angiogenic factor concentrations and birth outcomes (preterm birth and SGA) was further evaluated (Table 3). After adjustment, significantly lower concentrations of sFlt-1 and sFlt/PlGF ratio at the first trimester and significantly lower concentrations of PlGF at the second trimester were seen in SGA cases than in normal weight cases. No significant association between angiogenic factor concentration and preterm birth was observed.

**Table 3:** Association between biomarkers and adverse birth outcomes

Biomarkers	Crude coefficient	CI (95%)	*p*-value	Adjusted coefficient^a^	Adjusted CI (95%)	*p*-value
First trimester						
PlGF						
SGA	−0.006	−0.109 - 0.097	0.904	−0.023^b^	− 0.130 - 0.085	0.980
Preterm birth	0.054	−0.046 - 0.154	0.290	0.056^c^	−0.043 - 0.155	0.266
sFlt1						
SGA	−0.192	−0.384 - 0.001	0.051	−0.260^d^	− 0.432 - -0.088	0.003
Preterm birth	0.090	−0.100 - 0.280	0.350	0.064^d^	−0.106 - 0.233	0.458
sFlt1/PlGF Ratio						
SGA	−0.184	−0.415 - 0.048	0.119	−0.227^d^	− 0.437 - -0.016	0.035
Preterm birth	0.035	−0.193 - 0.264	0.760	0.002^e^	−0.207 - 0.211	0.986
Second trimester						
PlGF						
SGA	−0.096	−0.220 - 0.028	0.127	−0.117^f^	− 0.225 - -0.010	0.033
Preterm birth	−0.071	−0.193 - 0.050	0.248	−0.006^g^	− 0.113 - 0.100	0.909
sFlt1						
SGA	0.007	−0.147 - 0.161	0.930	0.048^h^	−0.121 - 0.216	0.576
Preterm birth	0.072	−0.082 - 0.226	0.920	−0.077^i^	−0.083 - 0.237	0.342
sFlt1/PlGF Ratio						
SGA	0.107	−0.097 - 0.306	0.291	0.162^j^	−0.038 - 0.362	0.111
Preterm birth	0.132	−0.067 - 0.331	0.193	0.013^k^	−0.193 - 0.218	0.903

*ART* Antiretroviral therapy, *CI* Confidence interval, *SGA* Small for gestational age

^a^Linear regression

^b^ Adjusted for gestational age at the date of test, maternal age, body mass index, parity, sex of the fetus and ART

^c^ Adjusted for gestational age at the date of test and ART

^d^Adjusted for gestational age at the date of test, body mass index, sex of the fetus and ART

^e^Adjusted for gestational age at the date of test, body mass index, ethnicity, parity, sex of the fetus and ART

^f^Adjusted for gestational age at the date of test, maternal age, body mass index, ethnicity, parity, and ART

^g^Adjusted for gestational age at the date of test, body mass index, ethnicity, parity, and ART

^h^Adjusted for gestational age at the date of test, maternal age, body mass index, ethnicity, parity, smoking, sex of the fetus and ART

^i^Adjusted for gestational age at the date of test, maternal age, smoking, sex of the fetus and ART

^j^Adjusted for gestational age at the date of test, maternal age, body mass index, ethnicity, parity, smoking and ART

^k^Adjusted for gestational age at the date of test, maternal age, body mass index, smoking, sex of the fetus and ART

## Discussions

In this cohort study, first and second trimester angiogenic factor concentrations are not significantly associated with ART exposure of any type and duration, nor are they associated with the timing of treatment initiation (pre-conception or during pregnancy). The biomarker levels observed among women living with HIV appear to be similar to those reported in HIV-negative women in other studies [44–48]. The strong association of these angiogenic factor concentrations with gestational age and other baseline data (BMI, ethnicity) is consistent with data from HIV-negative pregnant women [48, 49]. This explains why the significant differences between ART exposure groups in the unadjusted analyses were not confirmed in the multivariate analyses (Table 2). The association between angiogenic factor concentrations and adverse pregnancy outcomes is consistent with reports in HIV-negative women [27, 30] and in women living with HIV [37].

The absence of an HIV-negative control group in this study is a limitation that prevents us from assessing the impact of HIV infection itself on angiogenic factors and whether the infection itself can lead to dysregulation of angiogenesis. However, it seems unlikely that viral activity has much of an influence on angiogenic factor concentrations when we consider the similarity of levels in the untreated group (with a detectable viral load) compared to the treated one. The heterogeneity of ART regimens received by women is another limitation of our study (see Additional file 1: Table S1, which illustrates the different nucleoside reverse transcriptase inhibitors in the ART). As drug use was very rare in our cohort (< 1%), we could not evaluate the impact of this factor on placental angiogenic factors. However, repeated measurements in the first and second trimester increased the number of observations, giving us a greater statistical power. Measurements of the levels of angiogenic factors were all performed on the same day to minimize inter-assay variability. We also attempted to eliminate bias with a conservative method of adjustment for potential confounding factors.

Very few studies have explored the relationship between ART and the serum concentration of these angiogenic factors during pregnancy. Studies in oncology, however, have suggested anti-angiogenic effects for some PIs [20, 21]. The effects of PIs on placental vascular system formation and fetal development have to date only been examined in a mouse model [50]. Mice exposed to ART had significantly smaller fetuses and placentas compared to controls. Litter size and fetal viability were negatively impacted by exposure to two nucleoside analogs and two PIs at doses equivalent to human therapeutic doses. Although PlGF levels were unchanged, significantly lower levels of placental sFlt-1 were noted [19, 50].

Lower PlGF levels were reported in South Africa amongst pregnant women living with HIV, compared to uninfected pregnant women, whether or not they were preeclamptic [51]. However, the sample size in that study was small (27 HIV-negative women and 31 women living with HIV) and the authors did not specify if the subjects were receiving ART. By contrast, Govender et al. reported no relationship between HIV infection and angiogenic factors measured in the third trimester of pregnancy, but provided no details concerning ART exposure [25].

Even though our study is the first to report data on periconceptionnal and first trimester ART exposure, our results are consistent with those of a recent study in Uganda of 326 pregnant women living with HIV who began receiving ART in the second trimester. That study reported no significant difference in the levels of angiogenic factors according to the class of ART (PI vs non-nucleoside reverse transcriptase inhibitor (NNRTI) [37]. Similarly, another study of a cohort of 71 women whose ART was initiated after 26 weeks of pregnancy also found no association between the duration of ART exposure or changes in angiogenic factor concentrations [52]. The authors reported no significant changes in angiogenic marker levels after 1 month of ART, and no significant differences in serum concentration of sFlt-1 or PlGF relating to the type of ART (NNRTI versus PI).

To our knowledge, our study constitutes the first report on first-trimester angiogenic marker levels in a cohort of women who received early exposure to ART during pregnancy. Our results are consistent with reports on later ART initiation in pregnancy [37]. These finding needs to be confirmed by the study of circulating levels of other angiogenic biomarkers as well as direct study of the early placenta vasculature. If confirmed, even though angiogenic processes in the placenta are critical regulators of fetal growth and impact birth outcomes, the pathophysiological mechanism of the association between ART and preterm birth would unlikely be through an early and direct effect on placental angiogenesis. Immune restoration as a result of ART initiation is a hypothesis that needs to be explored [53]. Indeed, a study from the United Kingdom suggests that ART-induced immune reconstitution plays a central role in the pathogenesis of pre-eclampsia in pregnant, women living with HIV receiving ART. HIV infection could be associated with a low risk of preeclampsia and this risk restored to the expected values in women treated with ART [53].

## Conclusions

This study suggests that ART, whether PI-based or not, is not associated with the serum concentration of angiogenic factors PlGF and sFlt-1 in the first and second trimesters of pregnancy. There is also no significant association with duration of treatment or timing of treatment initiation (before conception or during pregnancy). These observations seem generally reassuring for the potential consequences of early ART use during pregnancy. Further studies are needed to confirm the safety of early ART exposure regarding placental angiogenesis and its implications for adverse pregnancy outcomes, especially considering the rapid evolution of ART guidelines.

## Supplementary information

**Additional file 1.** Table that illustrates the different nucleoside reverse transcriptase inhibitors in the ART.

## Data Availability

The datasets generated and/or analysed during the current study are not publicly available due to restrictions associated with anonymity of participants but are available from the corresponding author on reasonable request.

## Abbreviations

ART: Antiretroviral therapy
BMI: Body mass index
CMIS: Centre maternel et infantile sur le sida
ELISA: Enzyme-Linked Immunosorbent Assay
GEE: Generalized estimating equations
IQR: Interquartile range
NNRTI: Non-nucleoside reverse transcriptase inhibitor
PI: Protease inhibitor
PlGF: Placental growth factor
sFlt-1: Soluble fms-like tyrosine kinase-1
SGA: Small for gestational age
