# Supplementary material for: No association between early antiretroviral therapy during pregnancy and plasma levels of angiogenic factors: a cohort study
Source: BMC Pregnancy Childbirth. 2019 Dec 9;19:482. doi: 10.1186/s12884-019-2600-4 (PMC6902555; doi:10.1186/s12884-019-2600-4)
Supplement: Supplementary file 1 — Additional file 1. Table that illustrates the different nucleoside reverse transcriptase inhibitors in the ART. [file 12884_2019_2600_MOESM1_ESM.pdf]

Supplemental table 1. Nucleoside reverse transcriptase inhibitors in ART

**NRTI, *n* (%)**	**1st trimester** ***n*=109**	**2nd trimester** ***n*=153**
Zidovudine -Lamivudine	35 (32.1)	73 (47.7)
Zidovudine -Abacavir	2 (1.8)	4 (2.6)
Zidovudine-Didanosine	0 (0.0)	1(0.7)
Abacavir- Lamuvidine	33 (30.3)	36 (23.5)
Tenofovir - Emtricitabine	39 (35.8)	39 (25.5)

ART: antiretroviral therapy; NRTI: Nucleoside reverse transcriptase inhibitors
